# So, You Want to Have a Nanofab? Shared-Use Nanofabrication and Characterization Facilities: Cost-of-Ownership, Toolset, Utilization, and Lessons Learned

**DOI:** 10.6028/jres.125.009

**Published:** 2020-02-21

**Authors:** J. Alexander Liddle, Jerry Bowser, B. Robert Ilic, Vincent Luciani

**Affiliations:** 1 National Institute of Standards and Technology,Gaithersburg, MD 20899, USA

**Keywords:** cleanroom, cost-of-ownership, facility, nanofabrication, operation model, shared-use, utilization analysis

## Abstract

Nanofabrication/characterization facilities enable research and development activities across a host of science and engineering disciplines. The collection of tools and supporting infrastructure necessary to construct, image, and measure micro- and nanoscale materials, devices, and systems is complex and expensive to establish, and it is costly to maintain and optimize. As a result, these facilities are typically operated in a shared-use mode. We discuss the key factors that must be considered to successfully create and sustain such facilities. These include the need for long-term vision and institutional commitment, and the hands-on involvement of managers in facility operations. We consider startup, operating, and recapitalization costs, together with algorithms for cost recovery and tool-time allocation. The acquisition of detailed and comprehensive project and tool-utilization data is essential for understanding and optimizing facility operations. Only such a data-driven decision-making approach can maximize facility impact on institutional goals. We illustrate these concepts using the National Institute of Standards and Technology (NIST) NanoFab as our test case, but the methodologies and resources presented here should be useful to all those faced with this challenging task.

## 1 Introduction

The ability to fabricate and characterize micro- and nanoscale devices is an indispensable enabler for research and development across disparate scientific endeavors spanning engineering and the physical and life sciences. Typically, many different (and expensive) tools, which require specialized infrastructure, are needed to build and interrogate such devices. This combination of tools and infrastructure is far beyond the financial means of a single research group, division, or academic department to acquire and operate. Shared-use facilities are an effective way to overcome these obstacles, but assembling, supporting, and maintaining the necessary resources to create a state-of-the-art user facility are complex, challenging, and costly endeavors. In addition, it is essential to understand how such a facility supports the goals of the institution of which it is a part, and to optimize its configuration and operations for that purpose.

Given the scale of the investment required, the critical need for a long-term commitment, and the recurring costs involved in sustaining such a facility, it is highly desirable to target that investment carefully and understand if it is being utilized adequately. Unfortunately, the metrics available to commercial fabrication operations, such as wafer starts and yield, are not applicable to the extremely heterogenous mix of devices, processes, and analytical activities that take place in a full-featured research user facility. Here, we discuss the considerations that led to our current algorithms for charging, tool reservations, staffing, and training, and we provide the rationales that helped us to select the tool set for the nanotechnology user facility at the National Institute of Standards and Technology (NIST), the Center for Nanoscale Science and Technology (CNST). In addition, we developed a methodology and a comprehensive set of metrics that can be used to provide insight into user needs, and to determine facility capacity, to assess the level of utilization, and to identify areas for improvement. While the metrics will be different for facilities with different tool sets, user bases, and access models, we hope that the methodology presented here is flexible enough to be readily adapted for a variety of cases and to provide useful insights for those charged with operating nanofabrication user facilities. Most importantly, we hope to show how the collection of a comprehensive data set that captures both tool utilization data and user behavior provides a sound basis for making decisions. To that end, the CNST NanoFab developed, and has made available free-of-charge, a software platform, NanoFab Equipment Management & Operations (NEMO) [1], that provides access to detailed tool-by-tool and project-by-project usage data.

We begin with a short background to the CNST NanoFab, before discussing some of the economic aspects of fabrication (fab) operations. Since many of those considerations are driven by the toolset, we examine the relationship between the current and future needs of the users and the selection of the tools. We then introduce a variety of metrics to assess the level of facility utilization and illustrate how they can be used to suggest ways of maximizing the effectiveness of the operation.

## 2 Background

The CNST was launched in 2007 by consolidating some existing tools into a new cleanroom in order to provide access to a comprehensive suite of leading-edge fabrication and characterization tools necessary to make and measure nanoscale structures and devices in support of the NIST mission. Unlike university facilities, it is situated on a semiclosed campus, adding another level of complexity. At its inception, the NanoFab consisted of an 1800 m^2^ (19,000 sq ft) cleanroom space, of which 740 m^2^ (8000 sq ft) was ISO class 5 (100), with the remainder at ISO class 6 (1000). Approximately 46 m^2^ (500 sq ft) of standard laboratory space was set aside for postprocessing operations such as wafer dicing, wire bonding, and chemical-mechanical polishing (CMP). Later, the NanoFab expanded to include imaging and characterization tools, together with a soft-lithography laboratory and a suite of back-end-of-line (BEOL) tools, adding a further 350 m^2^ (3767 sq ft) of laboratory space, some of which required modification to achieve the necessary temperature and vibration specifications. The cleanroom class is relaxed compared to that for leading-edge manufacturing, yet it is appropriate for a research operation where lower device yields from particulate contamination are not a major driver. As a result, operating expenses in terms of power and high-efficiency particulate air (HEPA) filter replacement are also significantly reduced at these cleanliness levels. The initial toolset allowed for basic contact lithography, etch, high-temperature processing (furnaces and rapid thermal annealing [RTA]), and physical, plasma-enhanced chemical, and low-pressure chemical vapor deposition (PVD, PECVD, and LPCVD, respectively). In the years that followed, the baseline tools were replaced or upgraded to provide a path for processing 200 mm diameter wafers throughout the entire fab. This decision was primarily driven by a desire to make the fab compatible with many nontraditional or nonintegrated circuit (IC) industrial operations. A secondary consideration was that leading-edge technologies were becoming less available in tools that processed smaller diameter wafers. Lastly, the NanoFab supports a comprehensive suite of characterization and metrology tools and thus functions as an end-to-end imaging and fabrication facility.

**Fig. 1:**
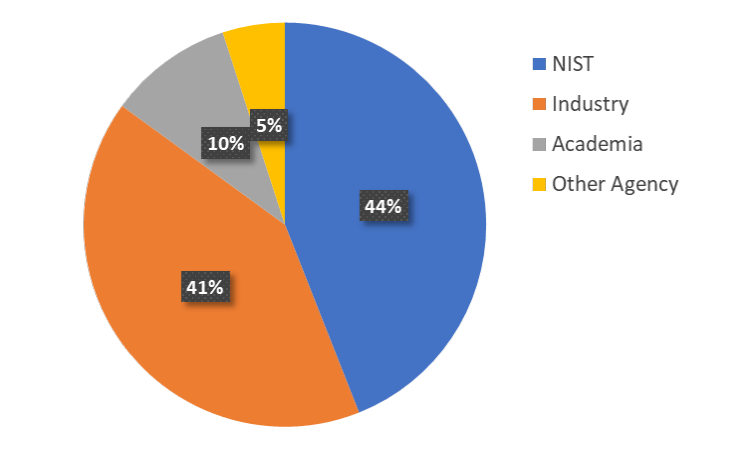
Distribution of projects between various user groups. In the 12 months between August 28, 2017, and August 28, 2018, the NanoFab supported 340 unique users and 198 projects.

The NanoFab supports a diverse range of projects and users, as shown in Fig. 1.

## 3 Toolset Cost, Cost-of-Ownership, Lifetime, and Recapitalization

**Fig. 2:**
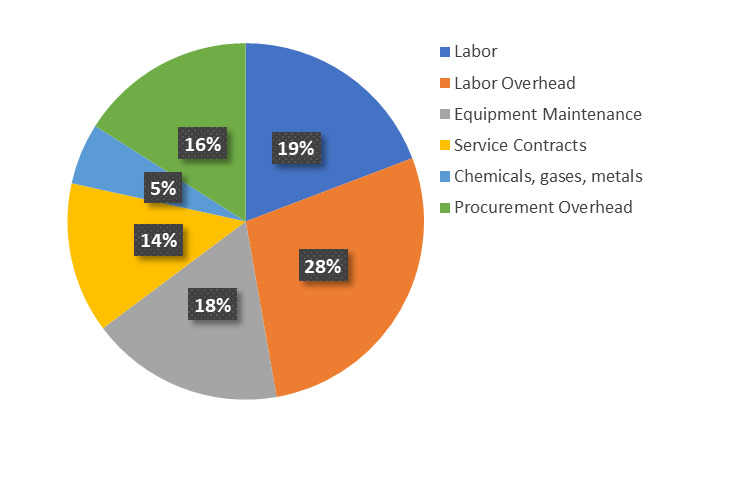
NanoFab operations budget, showing the distribution of the approximately $9M/year operating budget. Equipment maintenance costs include replacement parts such as vacuum pumps, planned maintenance kits, furnace tubes, *etc*.

As noted above, it is essential to accurately determine the startup costs and long-term cost-of-ownership of a fabrication and characterization facility. The magnitude of these costs typically means that high-level institutional commitment is required for such a facility’s ongoing success. Attempts at full cost recovery for facility use stifle research and innovation: Put simply, fee income derived from user projects is insufficient to offset operational costs, let alone new equipment acquisition and associated installation costs [2]. This institutional commitment will only be forthcoming if the facility performs effectively in supporting the institutional goals and maintaining the overall productivity and impact of the research enterprise, and the funding that it brings into the institution. This, in turn, depends on it being able to support competitive research and to work at the leading edge. A lack of commitment and the accompanying subcritical resourcing lead to a facility that underperforms and therefore becomes underutilized and ineffective while still acting as a major drain on institutional resources. Conversely, a sufficient long-term commitment can create a highly capable facility that enhances an organization’s ability to address complex, high-value projects. In addition, a facility with reliable longevity will tend to become an integral part of an increasing number of programs. Below, we examine the initial and ongoing costs associated with the CNST NanoFab. Again, while the details may vary widely between different facilities optimized for different purposes, we believe that the basic considerations are universal. Finally, we note that the NanoFab supports many programs that are critical to the NIST mission, making it somewhat different from other shared-use facilities. The primary goal of the NanoFab is to maximize NIST’s impact, and it enables NIST researchers to perform more and higher-quality research than would otherwise be possible. Its existence therefore benefits the institution as a whole, and it is therefore viewed as a “public good.” As such, it enjoys strong institutional support, both in terms of a substantial (≈ 50%) subsidy with regard to its approximately $9M/year operating costs (Fig. 2) and, perhaps uniquely, the existence of a separate budget that covers equipment recapitalization costs, *i.e*., depreciation and new capability acquisition. Full cost recovery is neither required nor expected.

A complete listing of the current toolset is provided in Table 1,^1^1 Certain commercial equipment, instruments, or materials are identified in this paper in order to describe the NanoFab configuration adequately. Such identification does not imply recommendation or endorsement by the National Institute of Standards and Technology, nor does it imply that the equipment identified is necessarily the best available for the purpose. with approximate acquisition pricing, installation, and maintenance/service contract costs. The percentages by process area of total acquisition, service contract, and installation costs are shown in Fig. 3. We have also included the relevant costs for the major support systems, such as deionized (DI) water, nitrogen (liquid and gas), acid neutralization, safety-related monitoring systems, solvent waste collection, access control, information technology, *etc*. Other important data are the anticipated tool or support-system lifetimes, which enable estimation of the recapitalization budget needed to maintain a facility at its current capability level. This should of course be adjusted annually by an appropriate factor to account for escalation in equipment costs. The recapitalization budget estimated in this way represents a lower limit. New fabrication and imaging technologies that bring new or improved capabilities are being developed constantly, and, to keep the facility at the leading edge, tool acquisitions beyond the current scope must be constantly evaluated. Occasionally, new tools may offer large enough improvements in support of institutional goals, such as user throughput, such that they allow multiple older systems to be retired, but this is, unfortunately, rarely the case.

**Table 1:** Breakdown of tool acquisition cost, useful lifetime, replacement cost (assuming an annual inflation rate of 3%, compounded over the tool lifetime), service contract cost, and depreciation (initial tool cost/lifetime). Figures are rounded to the nearest 1 000.

Area/Tool	Acquisition Cost ($)	Useful Life (years)	Replacement Cost ($)	Installation Cost ($)	Service Contract ($/year)	Depreciation ($/year)
**Deposition**						
Ion-beam/biased-target deposition system	3 650 000	15	5 687 000	80 000		243 000
ICP PECVD system	700 000	10	941 000	15 000		70 000
Atomic layer deposition	780 000	10	1 048 000	250 000	35 000	78 000
RGA for ALD	91 000	10	122 000	—		9 000
Sputter 1	199 000	15	310 000	25 000		13 000
Sputter 2	200 000	15	312 000	25 000		13 000
Sputter 3: Desktop	60 000	15	93 000	1 000		4 000
E-beam/thermal evaporator	199 000	15	310 000	25 000		13 000
E-beam evaporator	199 000	15	310 000	25 000		13 000
Tabletop thermal evaporator	59 000	15	92 000			4 000
Parylene deposition	125 000	10	168 000	10 000		13 000
RTA	265 000	10	356 000	104 000		27 000
Four-tube furnace (wet oxidation, anneal, nitride, poly silicon)	920 000	10	1 236 000	425 000		92 000
Four-tube furnace (wet oxidation, anneal, nitride, TEOS/LTO)	965 000	10	1 297 000	425 000		97 000
***Deposition total***	***8 412 000***		***12 282 000***	***1 410 000***	***35 000***	***689 000***
**Lithography**						
Optical i-line stepper	5 100 000	15	7 946 000	800 000	145 000	340 000
E-beam lithography 1	3 444 000	10	4 628 000	50 000	200 000	344 000
E-beam lithography 2	3 177 000	10	4 270 000	350 000	200 000	318 000
Contact aligner 1 (150 mm, front and back side)	400 000	15	623 000	5 000		27 000
Contact aligner 2 (front side)	400 000	15	623 000	5 000		27 000
Mask laser writer	1 080 000	10	1 451 000	20 000		108 000
Wafer laser writer	348 000		468 000	25 000		35 000
Nano-imprint lithography	49 000	15	60 000	5 000		7 000
UV–ozone cleaner for imprint	49 000	7	60 000	5 000		7 000
***Lithography total***	***14 047 000***		***20 129 000***	***3 525 000***	***545 000***	***1 213 000***
**Lithography Support**	Acquisition Cost ($)	Useful Life (years)	Replacement Cost ($)	Installation Cost ($)	Service Contract ($/year)	Depreciation ($/year)
Photolithography resist coat system	950 000	15	1 480 000	15 000		63 000
Aqueous developer/clean	275 000	10	370 000	15 000		28 000
Adhesion promoter (HMDS)	73 000	20	132 000	1 000		4 000
Deep UV photoresist stabilization system	57 000	10	77 000	1 000		6 000
Resist spinner/hotplate 1	44 000	7	54 000	1 000		6 000
Resist spinner/hotplate 2	44 000	7	54 000	1 000		6 000
Programmable hotplate 1	44 000	7	54 000	1 000		6 000
Programmable hotplate 2	44 000	7	54 000	1 000		6 000
Vacuum oven 1	2 000	20	4 000	1 000		—
Vacuum oven 2	2 000	20	4 000	1 000		—
Vacuum oven 3	3 000	20	5 000	1 000		—
Vacuum oven 4	3 000	20	5 000	1 000		—
***Litho. support total***	***1 541 000***		***2 293 000***	***36 000***	***0***	***125 000***
**Etch**						
Ion mill	819 000	15	1 276 000	125 000		55 000
Deep silicon etcher	750 000	15	1 168 000	25 000		50 000
Deep silicon etcher	378 000	20	683 000	100 000		19 000
Multipurpose ICP/RIE etcher 1	350 000	10	470 000	200 000		35 000
Multipurpose ICP/RIE etcher 2	350 000	10	470 000	200 000		35 000
Multipurpose ICP/RIE etcher 3	600 000	10	806 000	725 000		60 000
Multipurpose RIE 1	369 000	20	666 000	100 000		18 000
Multipurpose RIE 2	378 000	20	683 000	100 000		19 000
Silicon RIE	369 000	20	666 000	100 000		18 000
Downstream asher	450 000	10	605 000	15 000		45 000
Lift-off tool	275 000	10	370 000	15 000		28 000
HF vapor etcher	207 000	5	240 000	10 000		41 000
XeF_2_ silicon bulk etch	113 000	25	237 000	10 000		5 000
***Etch total***	***5 408 000***		***8 340 000***	***1 725 000***	—	***428 000***
**Metrology**						
Optical profilometer	250 000	10	336 000	—		25 000
Critical dimension microscope	200 000	20	361 000	1 000		10 000
Contact profilometer	187 000	7	230 000	1 000		27 000
Spectroscopic ellipsometer	175 000	7	215 000	1 000		25 000
Parametric test station	150 000	15	234 000	1 000		10 000
Mercury probe	318 000	10	427 000	1 000		32 000
Four-point probe	122 000	20	220 000	1 000		6 000
Stress measurement tool	76 000	20	137 000	1 000		4 000
Reflectometer	17 000	10	21 000	1 000		2 000
Contact angle goniometer	14 000	20	25 000	1 000		1 000
Optical microscope 1	19 000	20	34 000	1 000		1 000
Optical microscope 2	61 000	20	110 000	1 000		3 000
Stereo microscope	14 000	20	336 000	—		25 000
***Metrology total***	***1 353 000***		***2 375 000***	***6 000***		***122 000***
**Wet Processing**						
Spray acid tool	525 000	10	706 000	103 000		53 000
Spray acid tool	525 000	10	706 000	103 000		53 000
Spray acid tool	525 000	10	706 000	103 000		53 000
Solvent bench	165 000	5	191 000	65 000		33 000
Solvent bench	165 000	5	191 000	65 000		33 000
Acid bench	125 000	5	145 000	65 000		25 000
Acid bench	125 000	5	145 000	65 000		25 000
Spin rinse dryer 1	50 000	10	67 000	8 000		5 000
Spin rinse dryer 2 (CMOS)	50 000	10	67 000	8 000		5 000
Spin rinse dryer 3	50 000	10	67 000	8 000		5 000
Wet clean bench (dual RCA)	85 000	10	114 000	50 000		9 000
Wet clean bench (dual RCA)	85 000	10	114 000	50 000		9 000
Wet clean bench (CMOS RCA)	94 000	10	126 000	50 000		9 000
Wet etch bench (Si_3_N_4_)	85 000	5	99 000	50 000		17 000
Photomask process bench	85 000	5	99 000	50 000		17 000
Wet etch bench (KOH/TMAH)	94 000	5	109 000	50 000		19 000
***Wet processing total***	***2 833 000***		***3 651 000***	***921 000***		***369 000***
**Postprocessing**						
Wafer bonder	306 000	7	376 000	5 000		44 000
Flip-chip bonder	193 000	10	259 000	5 000		19 000
Wafer dicing saw	95 000	15	148 000	25 000		6 000
Wire bonder (Au)	25 000	15	39 000	1 000		2 000
Wire bonder (Al)	16 000	15	25 000	1 000		1 000
Critical point dryer 100 mm	50 000	7	61 000	5 000		7 000
Critical point dryer pieces	6 000	10	8 000	5 000		1 000
Scribe-and-break tool	150 000	15	234 000			10 000
Small scribe-and-break tool	20 000	15	31 000			1 000
Precision saw	6 000	5	7 000	1 000		1 000
CMP	341 000	10	458 000	30 000		34 000
Wafer cleaner	176 000	10	237 000	15 000		18 000
***Postprocessing total***	***1 384 000***		***1 883 000***	93 000		***144 000***
**Imaging & Characterization**						
Transmission electron microscope	2 991 000	10	4 020 000	300 000	153 862	299 000
Focused ion beam 1	1 003 000	20	1 812 000	50 000	70 000	50 000
Focused ion beam 2: EBSD	2 151 000	10	2 891 000	50 000	61 000	215 000
Focused ion beam 3: multiple gas injection system	2 049 000	10	2 754 000	50 000	61 000	205 000
Scanning electron microscope 1	1 400 000	20	2 529 000	50 000	30 000	70 000
Scanning electron microscope 2	800 000	10	1 075 000	116 000	46 000	80 000
Tabletop scanning electron microscope	200 000	7	246 000	1 000		29 000
X-ray diffractometer (XRD)	588 000	15	916 000	15 000	21 000	39 000
High-resolution AFM	300 000	10	403 000	2 000		30 000
Wafer-scale AFM	300 000	10	403 000	8 000		30 000
Tabletop grinder polisher	19 000	5	22 000	1 000		4 000
Tabletop sputter coater	54 000	5	63 000	1 000		11 000
Ion mill for TEM sample preparation	260 000	10	349 000	8 000		26 000
***Imag. & char. total***	***12 115 000***		***17 483 000***	650 000	***442 862***	***1 088 000***
**Facilities**						
DI water system	4 653 000	15	7 249 000	Installation included	180 000	310 000
High-purity nitrogen system	650 000	15	1 013 000	Installation included	205 500	43 000
Acid neutralization system	445 000	15	693 000	Installation included	25 000	30 000
Access control system	155 000	15	241 000	Installation included		10 000
Toxic gas monitoring system	2 370 000	15	3 692 000	Installation included	30 000	158 000
Process gas distribution system	550 000	15	857 000	Installation included		37 000
IT	75 000	5	87 000	Installation included		15 000
Solvent waste collection	15 000	3	16 000	Installation included		5 000
***Facilities total***	***8 913 000***		***13 848 000***		***440 500***	***608 000***
**Totals ($)**	**56 006 000**		**82 284 000**	**8 336 000**	**1 403 000**	**4 786 000**

ICP PECVD, inductively-coupled plasma plasma-enhanced chemical vapor deposition; RGA for ALD, residual gas analyzer, atomic-layer deposition; RTA, rapid thermal anneal; TEOS/LTO, tetraethylorthosilicate/low-temperature oxide; UV, ultraviolet; HMDS, hexamethyldisilazane; ICP/RIE, inductively-coupled plasma/reactive ion etch; CMOS, complementary metal oxide semiconductor; RCA, refers to cleaning chemistry developed by RCA; TMAH, tetramethylammonium hydroxide; EBSD, electron-beam backscatter detector; AFM, atomic-force microscope; TEM, transmission electron microscope; DI, deionized; IT, information technology.

**Fig. 3:** Percentage of cost by process area for acquisition, service contract, and installation costs. **Table:** 

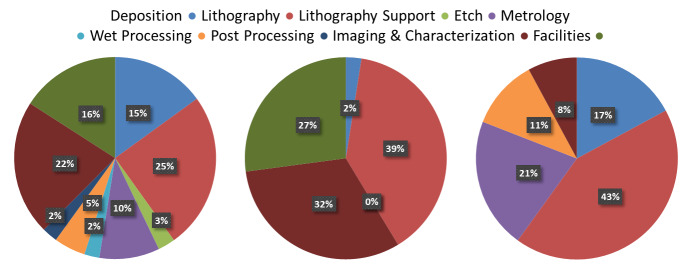		
**Acquisition Cost ($56M)**	**Service Contracts ($1.4M)**	**Installation ($8.4M)**

After a decade of acquisitions, the CNST NanoFab has a state-of-the-art toolset. During this time, the emphasis has been on developing both outstanding capabilities and maximizing user productivity. The continuing challenge is to ensure that the toolset is maintained at this high level of availability and performance. Addressing this challenge is essential to keeping the NanoFab relevant and therefore effectively and efficiently used. Subsequent tool acquisitions are thus intended primarily to replace systems that are reaching the end of their useful life, with judicious addition of new capabilities, as user demand dictates, and as funding for both staff and procurement of tools and service contracts permits.

**Fig. 4:**
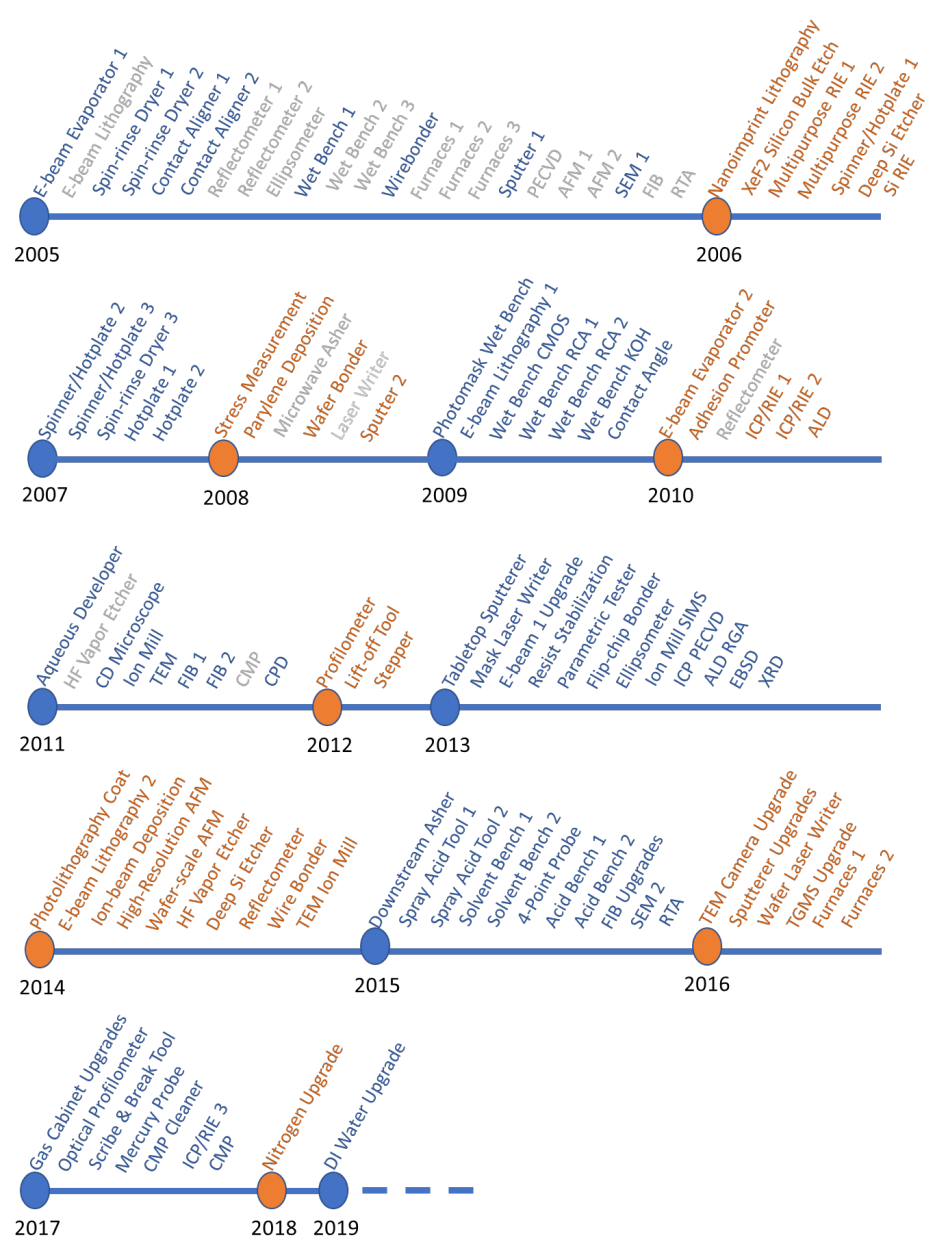
Timeline showing tool and infrastructure installations, upgrades, and replacements. Items in gray have been retired. PECVD, plasma-enhanced chemical vapor deposition; AFM, atomic force microscope; SEM, scanning electron microscope; FIB, focused ion beam; RTA, rapid thermal anneal; RIE, reactive ion etcher; CMOS, complementary-metal-oxide-semiconductor; ICP/RIE, inductively coupled plasma/reactive ion etcher; ALD, atomic layer deposition; CD, critical dimension; TEM, transmission electron microscope; CMP, chemical-mechanical polishing; CPD, critical-point dryer; TGMA, toxic gas monitoring system; SIMS, secondary-ion mass spectrometer; RGA, residual gas analyzer; EBSD, electron-beam backscatter detector; XRD, X-ray diffractometer; DI, deionized. An initial tranche of tools was acquired from smaller, preexisting facilities on the NIST campus, and so the start date for some tools predates the establishment of the CNST.

Figure 4 shows the timeline for major tool and infrastructure installations, upgrades, and replacements. Recently, and at considerable expense, a number of wet benches, the pure nitrogen delivery system, and DI water system had to be replaced. In a lesson learned, these replacements were necessitated by cost-saving measures implemented during initial construction, which led to the installation of general-purpose wet benches, as opposed to cleanroom-qualified ones, and to nitrogen and DI water systems that were not sized for a fully equipped cleanroom. The cost to remedy these decisions has significantly exceeded the cost savings during construction, even before accounting for the associated downtime and disruption to research programs. In addition, as a significant cost-saving measure, and to speed tool installations, we invested in orbital welding equipment and associated training, including line testing and certification, so we have in-house capability to run both gas and DI water lines. We are now able to install double-walled toxic gas lines with *in situ* monitoring and automatic shutoff systems. The cost to acquire orbital welders for gas and DI water lines was $60 000 and $20 000, respectively, and training for six staff members was $15 000. This may be compared against a single quote from an external vendor for a 60 m run of gas piping of $200 000.

While we expect the pace of acquisitions will remain relatively constant now that steady state has been reached, the time and cost for installations will be reduced, since many of the facilities required for replacement tools will already be in place. We discuss the evolution of the toolset from the initial cleanroom concept to its current status in the “Toolset Selection” section below.

## 4 Intellectual Property

The operation of a user facility inevitably leads to concerns regarding intellectual property, particularly in the context of a government-operated one. We address these concerns through our facility user agreement. This permits both proprietary work (charged at the full cost recovery rate) and nonproprietary work, which is eligible for a reduced rate. Proprietary projects allow users to maintain full control over any intellectual property. Nonproprietary projects enable users to interact with staff to solicit advice, troubleshoot problems, and get help with process development, and to share information. In the event that intellectual property is generated *via* a user-staff interaction, ownership of the idea falls along standard lines: A user invention belongs to the user, one that is joint is jointly held, and one that is devised by staff belongs to NIST. Jointly held and NIST-owned intellectual property can be licensed.

## 5 Facility Governance

Some type of formal process is required to gather input and ensure that the facility is meeting the needs of its users. There are typically short-term operational concerns and long-term strategic issues that need to be addressed. We have chosen to have two advisory groups. The first consists of actual users of the NanoFab, and it provides input and feedback concerning the day-to-day operations of the NanoFab, enabling continuous improvement in operational efficiency and the NanoFab’s ability to respond to user needs. The second consists of a selection of senior advisors, drawn from the institution’s staff, who provide input on strategic scientific and technological directions for the NanoFab to enable it to most effectively serve the user community and maximize impact over the long term. Both groups provide advice and input on tool selection. It should be noted that the final authority and responsibility for the NanoFab operations and toolset reside with the NanoFab manager, since there can be only one captain for the ship.

## 6 Staffing

The other major expense associated with a nanofabrication user facility is the cost of the staff necessary to maintain and operate it safely and effectively. The number of staff members and the appropriate mix of skill sets depend on the mission of the facility, the toolset, and the nature of the institutional support infrastructure. The CNST NanoFab’s principal role is to support the NIST mission, and it therefore must enable scientists to develop and deploy measurement standards, devices, and systems that are constantly advancing the state of the art. Given the large and leading-edge toolset, it is essential that all the instruments are reliable and maintained at their maximum level of performance. Some fabs rely on, for example, research staff or heavy users, who have a mix of other responsibilities, to maintain tools and processes. Such a co-op model can work for a small and fully engaged group of users, but it does not scale well. Given the number and diversity of projects that our NanoFab handles, we have chosen to support the fab operations with dedicated staff with the intention of maximizing the availability of tools operating at their optimum performance levels. Under this model, we employ several equipment technicians, and we operate in a manner such that NanoFab users are not expected to be involved in tool repair, maintenance, or training, and we have therefore chosen not to adopt a “super-user” model in which users are allowed to train other users. This model, may, however, be appropriate when specialized tools, or processes that are critical to a major program, are involved. In our case, having maintenance and repair expertise within the NanoFab helps to reduce the number of service contracts required, particularly for systems that do not require replacement parts from the manufacturer. For those that do require expensive replacement parts, such as electron-beam lithography tools that periodically need new sources, service contracts may be cost-effective. This in-house expertise also allows us to undertake complex equipment installations, helping to reduce costs. As the value of having equipment technicians on staff has been demonstrated, we have increased their number from two to four over the past decade. We also evaluate the cost-effectiveness of all service contracts and have eliminated a number as our expertise in maintenance and repair has increased.

Ensuring that the tools are operating optimally also requires that they be exercised and monitored by the staff *via* a set of baseline processes. In addition, given the constantly changing mix of projects, and the varying levels of expertise of our users, it is critical for the staff to be able to assist users by developing novel processes, and by providing training when needed. We therefore also employ a number of process engineers who have deep expertise in their areas, have sufficient breadth to deal with complex process integration issues, and who also have a talent for working with users from a wide variety of technical backgrounds (Table 2). Initially, the NanoFab management had primary responsibility for overseeing the staff and arranging user projects. However, in a lesson learned, it became apparent that if managers do not spend significant time in the cleanroom, then they can lose touch with the day-to-day operational aspects of the facility. We therefore decided that every manager needs to be personally responsible for specific tools, processes, and users in the NanoFab. This has dramatically improved communication and improved time-to-resolution for any problems, since managers now have firsthand insight into them. As an added benefit, managers have an accurate picture of how long training and maintenance tasks take, and they are in the NanoFab for extended periods of time so they can evaluate staff performance directly. We also found that a rigid separation between the duties of maintenance technicians and process engineers was causing significant delays in tool repair. To resolve this problem, the maintenance staff members are now trained in basic process knowledge, providing a path for career advancement, and the process engineers can resolve common tool failures. This ensures that tools are not released to users after repair until they are properly qualified, *i.e*., the baseline processes meet specification, and that users experience only minimal delays when tools fail. In addition, all staff have secondary responsibilities, and they are trained on multiple tools to ensure that there are no gaps in coverage when staff members are unavailable. This also provides operational resiliency in the event of staff turnover. Lastly, we strongly encourage staff to keep their skills current by taking short courses at conferences and attending vendor workshops.

**Table 2:** NanoFab staffing breakdown. All facility managers now also have process engineering responsibilities, as indicated by the color coding. The total number of full-time staff is 20.

**Position**	**Primary responsibility**	**Secondary responsibility**
NanoFab manager	Lab operations and laser lithography	Physical vapor deposition, lithography, CMP, metrology
Assistant manager	Lab operations, ICP reactive ion etch, and evaporation deposition	RIE and ALD
Assistant Manager	Lab operations	Thin films, etch, and maintenance
Group administrator	Administrative (property, travel, ordering, etc.)	
User office coordinator	User office	
User office coordinator	User office	
Lithography engineer	E-beam, stepper, and laser lithography, wet chemistry	Lithography, metrology
Lithography engineer	E-beam lithography, PECVD, metrology	Furnaces, lithography, metrology
Lithography engineer	Stepper lithography, metrology, wet chemistry, lab safety	Lithography, metrology, physical vapor deposition
Process engineer	Soft lithography, wet chemistry	PECVD and metrology
Etch engineer	ICP reactive ion etch, atomic layer deposition	Metrology and etch
Thin-films engineer	Sputter deposition, ion mill, metrology, CMP, BEOL	Physical vapor deposition, metrology, RIE
Thin-films engineer	Furnace, reactive ion etch	Metrology and RTA
Thin-films engineer	Sputter deposition, equipment maintenance	Physical vapor deposition
Microscopist	SEM, nanoparticles, metrology, sample preparation	Microscopy
Microscopist	TEM, TEM sample preparation	Microscopy
Microscopist	FIB	Microscopy and soft lithography
Engineering technician	Equipment maintenance, equipment installation, laboratory facilities	Physical Vapor Deposition
Engineering technician	Equipment maintenance, TGMS	RIE
Engineering technician	Equipment maintenance	Laboratory facilities
Equipment technician	Equipment maintenance	Laboratory facilities

ICP, inductively coupled plasma; RIE, reactive ion etch; ALD, atomic layer deposition; PECVD, plasma-enhanced chemical vapor deposition; CMP, chemical-mechanical polishing; BEOL, back end of line; RTA, rapid thermal anneal; SEM, scanning electron microscopy; TEM, transmission electron microscopy; FIB, focused ion beam; TGMS, toxic gas monitoring system.

Since the NanoFab operates as a fee-based facility, a significant information technology (IT) infrastructure is necessary to onboard new users, control cleanroom and tool access, schedule safety, tool and process training, support all aspects of billing, and track usage data and NanoFab-related outputs and impacts. We therefore also employ IT staff on an as-needed basis.

Finally, we note that our current approach is to have the cleanroom fully staffed on weekdays during normal, *i.e*., first shift hours; to have one or two staff members present for the second weekday shift, *i.e*., 16:00 h to 24:00 h; and to allow access with a buddy system outside those hours. It is worth considering if it is most effective to have the majority of the staff present during normal, *i.e*., first shift hours, or to have more uniform staff coverage during facility operating hours in terms of maximizing the facility output. In our NanoFab, with the current staffing profile and NIST campus access policies, we find that cleanroom occupancy peaks in midafternoon and tails off significantly after 20:00 h. However, in a university setting, where campus is always accessible, there may be more opportunities to promote different usage patterns.

## 7 User Facility Data Acquisition

As noted above, the CNST NanoFab has developed a unique system (NEMO) for monitoring and controlling access to the entire facility, and to every major tool within the facility. Each tool has a list of authorized users and is available only to those authorized users. NEMO serves as a reservation system that allows the reservation policy to be customized for each tool, and it provides usage data (including user, staff, maintenance, and offline hours) as a function of each tool, project, and individual facility user, data which are then passed to the billing system. The data are available in real time, enabling a quick count of which staff and users are in which space, which can be important for safety. It also serves to disseminate information, such as specific tool outages, maintenance, or facility shutdown notices, to the staff and users. For example, if a user discovers a tool malfunction, they log their observations in NEMO and mark the tool as shut down, alerting the staff to the problem. NEMO enables the staff to log details of the problem and communicate progress towards its resolution to the users. Each tool therefore has a detailed history associated with that specific tool. These data enable common or recurring problems to be identified and, ideally, preemptive action to be taken to minimize tool downtime. This can involve building an inventory of spare parts or developing engineering solutions to eliminate common tool failure modes or damaging user errors.

## 8 To Charge or Not to Charge?

Shared-use facilities typically represent a major institutional investment, and, given the high cost of operation, there is substantial pressure to recoup those operational costs as fully as possible. This can frequently have negative consequences at the global, institutional level, as users attempt to optimize their resources locally by reducing their activity to control budgets, resulting in both a fall in output and a decrease in productivity. The natural response is for the facility to maintain revenue by increasing the costs for the remaining users, and the ensuing vicious circle has resulted in the eventual collapse of some shared-use operations. One of the most vexing questions that confronts the managers of shared-use facilities is therefore how to cover costs and whether or not to charge. Even in the event that the financial resources exist to support the facility completely, it may make sense to charge for access in order to avoid the “tragedy of the commons” [3]. Conversely, charging for access presupposes that “hours of facility use” represent a resource in short supply, and that demand should be controlled by market forces. This is unlikely to be the case in all but the busiest research cleanrooms, and it may therefore make sense to develop schemes to encourage rather than constrain use. In addition, some mix of models may be appropriate, for example, if only one or two tools are bottlenecks. The financial model that is most appropriate depends on the number, type, and longevity of users, as well as the organizational goals.

These goals may be to identify certain key technology areas that the facility will champion and in which it will develop expertise. It may be to maximize support for the members of that organization and enable them to compete effectively for external funding. It may also have to serve a significant educational role. Alternatively, it might be to provide the widest access possible to external users. Since the financial model has a dramatic impact on how a facility is used, it is critical to align the model, and the behaviors it encourages or discourages, with the organizational goals. For example, a shared resource that supports a small, closed group of expert users may be best served by a simple co-op algorithm in which each user “buys in” or shares the cost at a level commensurate with their usage and supports the operation by maintaining tools and processes. The effective shared ownership and strong community lead to a self-policing operation [4]. On the other hand, a facility such as CNST, which is open, which serves a large number of users with a heterogenous mix of needs, with varying levels of expertise, and in which there is a constant influx of new users, would need a system of imposed controls to ensure equitable allocation and sustainable use of the resources. One part of this system is a mechanism that aligns an individual user’s cost-benefit incentives with those of a sustainable operation. The simplest way of doing this is to charge for access. There are several ways of applying access charges. One approach, popular with users, is to have some type of cap, which can be based on individual or project usage (or both), and which can involve limits that refresh annually, or on shorter timescales. Once the cap is reached, charges can decrease by some large percentage or be reduced to zero. However, a fee-cap algorithm tends to lead to abuses. In the case of the fees falling to zero once the cap is reached, the resources are effectively free, and we are back to the “tragedy of the commons.” A rate reduction once a cap is reached avoids this but tends to privilege heavy users over others. This may or may not be desirable depending on the user base and goals of the facility. For instance, if heavy users are also expert users, then a lower rate or cap for those individuals may be beneficial, since they will be more productive, will develop more and increasingly robust processes, will tend to use equipment in a way that minimizes the need for repairs, will require less staff assistance, and will help less-expert users. Alternatively, it can lead to a situation in which the facility is effectively dependent on a very small number of customers. If one of those leaves, then much of the justification for supporting the facility disappears, and it becomes unsustainable. For our NanoFab, we have chosen to adopt a straightforward model in which cleanroom and tool access are fee-based, with the underlying fee structure based primarily on the full cost recovery rate for each individual tool. While it would likely be impossible to recover the fully loaded rate, it is a useful starting point when setting rates. The hourly cost recovery rate for tools is calculated by dividing the operating costs (staff time, service contracts, consumables) by 2 250 h, which represents a working year (9 hours per day, 250 working days). An hourly cleanroom access charge, in addition to the individual tool charges, covers gowning, chemicals, and other incidental expenses. Rates for some tools are adjusted in order to make sure that some of the more expensive, but critical tools are not priced out of range for the majority of users, or to modulate demand for heavily subscribed tools. The rationale for this approach is that it provides a simple and transparent system for controlling usage and distributing the cost of the facility equitably to the users.

Another, often contentious question is whether or not to charge for training. Initially, we did not charge for training in order to minimize the barrier to becoming a NanoFab user. However, in a lesson learned, we quickly discovered that the NanoFab was being used as a place to park students and postdoctoral researchers when their advisors were unavailable. As a result, we now charge for the cost of the tool time plus that of the staff time needed for training. Our subsequent experience, which is consistent with that of many other shared-use facilities, is that some cost associated with training helps weed out the “fab-curious” and ensures that only those actually intending to use the fab take up staff time. In addition, the fact that training is being provided to paying customers ensures that staff members work to develop good training materials and protocols.

## 9 Reservation Algorithm

Another key component in ensuring sustainable and efficient operation is the algorithm used to manage tool reservations. In an ideal world, every user’s process flow would work flawlessly, and the time taken for each step would be known in advance. While this is true in a production environment, in a research fabrication environment, this is rarely the case. In this situation, it is important to devise rules for tool reservations that maximize tool usage and user productivity. In addition, given the very different nature of operations between, say, an LPCVD furnace (batch process) and an etch tool (single wafer), the rules need to be optimized on a tool-by-tool basis. We chose to implement several features in our reservation system that would help to minimize unused tool time, allow users adequate access to complete process steps even when they take longer than anticipated, and provide sufficient flexibility to allow users to directly resolve conflicts without the need to involve staff. To achieve these goals, our system features interlocks on the primary tools [5] that prevent tool usage without a login from an authorized user, and we introduced charges for unused reservation time, releasing reservations if work has not begun within, *e.g*., 30 min of the nominal start time, and limiting both the length of time and how far in advance a tool may be reserved. Each parameter is configurable by tool, including the missed reservation charge, how long to wait before automatic cancelation, the maximum length of reservation time, and how far in advance a reservation can be made, and these are constantly refined as we accumulate user statistics and feedback. Charging for unused reservations naturally curtails the tendency for users to book tools “just in case” and encourages careful planning of workflows. Reservations may be released at the last minute without incurring a penalty, as can the remainder of a reservation if work is completed early. While this is not ideal, it does offer an opportunity for users whose work is progressing faster than anticipated to “jump in” and make use of time that would otherwise go to waste and encourages community-minded behavior, where users keep each other informed about their progress so that they can trade reservations as circumstances dictate.

**Fig. 5:**
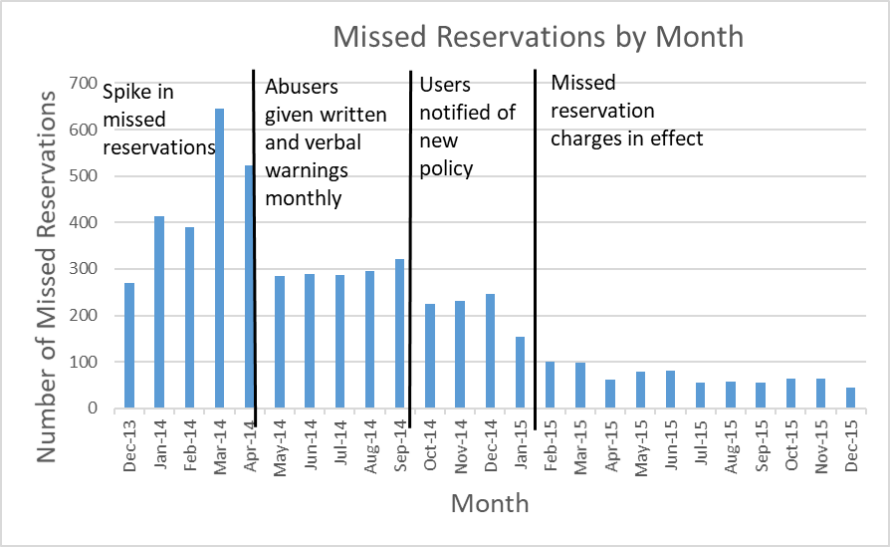
Time series of missed reservations, illustrating the positive effect of introducing a charge for missed reservations.

Our current reservation policy was a significant lesson learned and was implemented due to a spike in missed reservations in the winter of 2014 (Fig. 5) that caused productivity problems for our users. A few users started making “just in case” reservations that limited access to several key tools, including our sputter deposition systems and field-emission SEM. In response, the user base at large followed suit, resulting in a spike of approximately 650 unused reservations in one month. The immediate response was to use the data from NEMO to identify the abusers and start giving verbal and written warnings on a monthly basis. This was successful in lowering the monthly missed reservation to our historical norm of around 300. However, this solution had two significant problems. First, it was a manual process to analyze the data, contact each abusive user, and track reservation abusers month over month to decide if penalty escalation was needed. Second, and most importantly, the missed reservation time was not canceled, so the tools were blocked from other users accessing unused time slots. Therefore, we updated our policy to have NEMO automatically cancel a reservation if a user does not login within the first 30 min and to charge that user a missed reservation fee based on the cost of using the tool and how easily another user could use the open time. For example, any user with a sample ready to etch can use the reactive ion etchers; however, a sputter tool will likely need targets to be configured, so a missed sputter reservation has a higher impact than a missed etch reservation. The gradual introduction of the policy meant that the majority of users modified their behavior without the need for any sanction, though a handful of users were subject to missed reservation charges once the policy was fully in effect. The successful outcome of this approach suggests that reservation policies can be used in conjunction with charging to encourage optimal user behavior.

## 10 Toolset Selection

The basic process steps in device fabrication—deposition, lithography, etch, and characterization—provide the starting template for designing the tool mix in a fab. However, the diversity of materials, devices, research focus areas, users, and available resources determines the exact complement of tools. Determining the optimum toolset depends upon having a good understanding of the current and future user base. In the initial phases of operation, the facility manager must make a best guess as to the best starting toolset based on a synthesis of the organizational goals and input from likely users. Evolving from the starting configuration to the optimum toolset depends upon having a strong sense of and communication with the user base, which may itself evolve based on the availability of certain tools and the development of areas of expertise in the fab. The institutional vision and goals must also be followed. In addition, tools that look good on paper may not perform as advertised. Dealing with such situations is painful and costly, both financially and in terms of the impact on project timelines. An important lesson learned has been that multiple rigorous reviews of specification documents are necessary prior to any procurement. In addition, extensive communication with peer facilities is essential, *e.g*., *via* the University–Government–Industry micro/nano Symposium (UGIM) and mid-Atlantic facilities group, drawing on the wisdom and experience of the crowd to identify potential problems.

Figure 6 below shows the fraction of activity in the six principal research areas supported by the NanoFab in terms of number of projects, number of users, cleanroom activity (user hours and tool hours), and cleanroom revenue.

All five views are necessary to give a balanced picture of the demands placed on the NanoFab. We note that it is not always possible to cleanly assign a project to a single category, and that the choice of categories is somewhat arbitrary and facility dependent, but with these caveats in mind, useful information can be derived from this data set.

**Fig. 6:** Distribution of projects, users, NanoFab revenue, cleanroom hours, and tool hours by project category. The statistics were compiled from data taken between August 28, 2017, and August 28, 2018. MEMS, microelectromechanical systems. SRM, standard reference material. **Table:** 

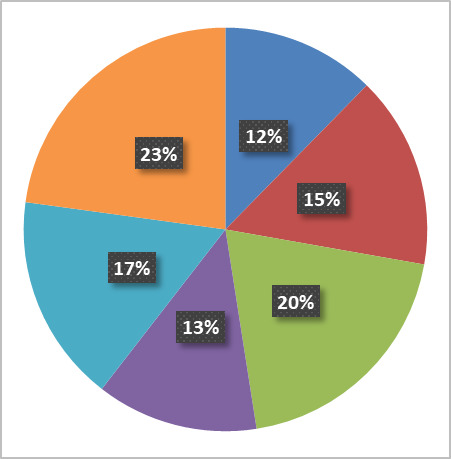	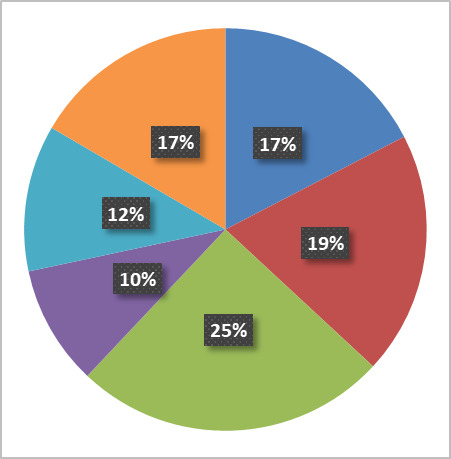
**Projects: 162**	**Users: 374**
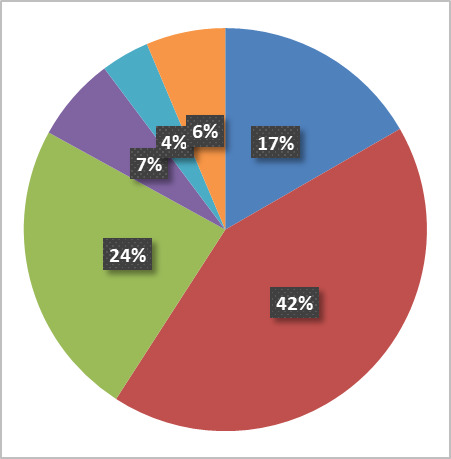	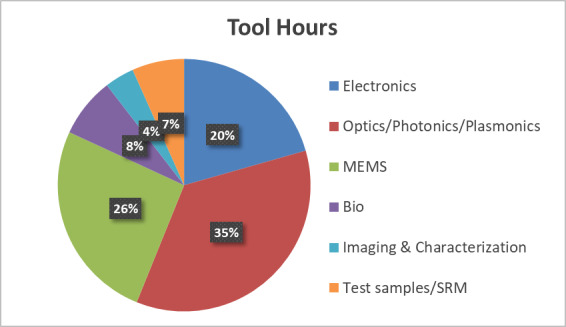
**Revenue: $4.38 M**	
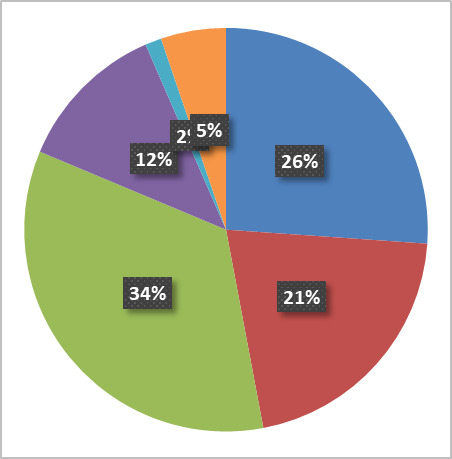	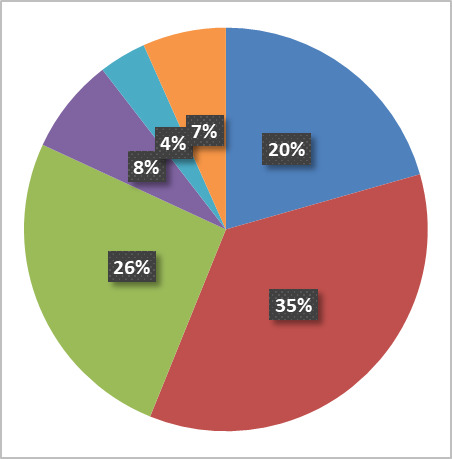
**Cleanroom Hours: 27263**	**Tool Hours: 33407**

For example, by examining these charts, we can see that, while the numbers of projects and users are relatively evenly divided between the categories, projects in the optics/photonics/plasmonics category make up the lion’s share of spending by users (Fig. 6). This is not surprising, given the need to fabricate large-area, sub-wavelength structures that require long write-times on high-cost electron-beam lithography systems. The production of microelectromechanical systems (MEMS) typically requires many process steps, extensive process integration, including metrology, and therefore a great deal of time in the cleanroom. Conversely, the fabrication of test structures often requires nothing more complex than the deposition of a thin film, so, even though many projects and users fall into this category, there is not a correspondingly large number of cleanroom hours or dollars spent. Similarly, many of the characterization projects require little time to provide the needed information. In addition, the total demand on characterization tools is not well captured using these metrics because so much fabrication activity includes characterization. However, the tool utilization data do provide an accurate view of the demand for these tools. This serves to emphasize once more the importance of examining all the available data to ensure that the optimum strategic decisions are made.

Below, we explain the rationale behind the selection of the major tools in each area of the NanoFab [6]. As background, we note that, given the wide availability of complementary-metal-oxide-semiconductor (CMOS) device fabrication foundry services, we determined that it would not be cost-effective or efficient to maintain a dedicated CMOS path through the fab, but we have instead chosen to allow a large number of different material systems into the NanoFab, including Si, SiC, GaAs, InP, diamond, CdSe, *etc*. While this allows us to support a broad range of projects, it does present challenges in maintaining adequate process segregation to ensure process stability and robustness. We note that all materials and chemicals used in the fab must be vetted and approved by the NanoFab manager for specific processes and tools. We expand on this in the following sections.

### 10.1 Deposition

Almost every fabrication process begins with the deposition or growth of one or more active layers, which may comprise metals, dielectrics, or semiconductors. A fab therefore needs a selection of deposition systems. Our NanoFab has received requests to deposit materials such as ^10^B, ^7^Li, and Gd, which support the nearby NIST Center for Neutron Research (NCNR), as well as the more typical materials. The unusual materials are mission-critical for NIST and must therefore be made available. While this particular selection may be unique, many fabs are required to support materials and processes that are not necessarily in high demand, but that are essential to some major local program. Such unavoidable “market distortions” must be carefully planned for and supported.

#### 10.1.1 Physical Vapor Deposition (PVD)

PVD is the most heavily subscribed part of the NanoFab, with approximately 40 different materials available. We therefore maintain several deposition systems (two electron-beam/thermal evaporators, two sputter systems, and one ion-beam–assisted deposition tool), both to deal with the number of materials and to provide adequate capacity to meet demand. Basic depositions can be conducted using electron-beam or thermal evaporation, while sputter deposition provides access to a larger selection of materials, together with the ability to exert some control over film morphology and stress. Both sputter systems have been equipped with load locks to maximize user productivity. The ion-beam/bias-target deposition system is a specialty tool capable of producing ultrahigh-purity, nanometer-scale continuous films of metals and dielectrics, with exceptional control over surface and interface morphology, and it permits users to build complex multilayer films that are essential to advancing our programs in quantum tunneling–based devices, nanomagnetics, plasmonics, and metamaterials.

#### 10.1.2 Chemical Vapor Deposition (LPCVD and PECVD)

Low-pressure chemical vapor deposition (LPCVD) is essential for producing high-quality films of amorphous Si, polycrystalline Si, doped polycrystalline Si, stoichiometric and low-stress silicon nitride, and low-temperature oxide (LTO) silicon dioxide. These materials are standard to most fabs, but we developed protocols to enable their deposition on nonstandard substrates, including wafers that already have some specialized metallizations, such as Pt. This protocol arose from a need to introduce electrodes into monolithic nanofluidic devices that are fully integrated on-chip. The fluidic channels are formed by encapsulating a chromia layer between two LPCVD SiN films. LPCVD is required for deposition since it produces the highest quality SiN. The Pt electrodes are therefore patterned before the second nitride deposition step.

Since this was not one of our baseline processes, we consulted with the tool owner and the tool manufacturer and did a considerable amount of background research. The key question in this instance was: Will the use of Pt in this process flow contaminate any tools or disrupt any existing processes? Preliminary experiments showed that Pt on SiN could pass through the standard RCA cleaning processes, and literature reviews indicated that it would not be mobile in the LPCVD tool. In addition, the Pt would be encapsulated by the inert, refractory chromia layer. The NanoFab manager therefore decided that the risk was acceptable.

Ultimately, it is the responsibility of the NanoFab manager to determine whether a novel process is worth exploring. If so, then the researcher and NanoFab tool owner work to develop that process.

#### 10.1.3 Furnaces

Along with the LPCVD deposition tubes, the NanoFab maintains tubes that operate at high temperatures for wet and dry oxidation and annealing. Very high temperature operation is enabled by the use of SiC furnace tubes. Rapid thermal processing up to 1500 °C is also available.

#### 10.1.4 Atomic Layer Deposition (ALD)

Atomic layer deposition results in highly conformal coatings, even over very high aspect ratio structures. Metal and dielectric films can be deposited with approximately monolayer control over thickness. The range of precursor materials is continually expanding, with standard, thermal, and remote plasma processes, including SiO_2_, HfO_2_, HfN, Al_2_O_3_, AlN, and Pt.

### 10.2 Lithography

Lithography is a critical step in device fabrication and consists of pattern design as well as the creation of a pattern in a physical resist layer. The link between throughput and feature size, as well as the diversity of applications, means that various tools are needed.

#### 10.2.1 Design 

The NanoFab provides both commercial design software as well as the in-house–developed NanoLithography Toolbox [7] to users. This is freely available and lowers the barrier to entry for new fab users and enables rapid design iteration to occur during fabrication runs.

#### 10.2.2 Electron-Beam Lithography

Leading-edge devices frequently require nanoscale features and alignment and overlay that can only be produced with high-resolution, dedicated (*i.e*., not SEM-based) electron-beam lithography. Compared to other lithography systems, these tools have relatively low throughputs and are in high demand. We therefore offer users access to two identical tools capable of producing features down to ≈ 10 nm. Having two available tools not only helps to satisfy demand, but it also enables us to support a mix of long and short write-time projects without delaying users. Finally, having redundancy in this area mitigates any adverse impact on projects if one tool goes down.

#### 10.2.3 Stepper Lithography

While steppers are normally associated with high-throughput production environments and may not at first seem appropriate for a research operation, the overlay accuracy they provide enables complex, multilevel devices. The addition of backside alignment and a capacity to accommodate thick wafers support the fabrication of sophisticated MEMS devices. The time to set up a job on the stepper can take far longer than the job run time. To avoid this, and maximize stepper utilization, we offer users a separate computer system for job setup. In a lesson learned, we found that users were attempting to save money by using low-quality soda-lime glass masks suitable for the contact printer in the stepper. This resulted in masks getting jammed in the stepper often, with each instance disabling the tool for two days. To eliminate this, we now only allow high-quality quartz masks in the NanoFab that meet the tighter mask blank flatness requirements associated with the stepper. Our choice of i-line (365 nm) as the stepper’s operating wavelength was driven by the desire to have low-cost, robust processes available. While deep-UV tools with 248 nm or 193 nm wavelengths offer better resolution, they are more expensive, have higher operating costs, and require more expensive resists. In addition, processing those resists is more complex, and they can be adversely affected by airborne base contamination, which means that expensive air filtration systems are needed.

#### 10.2.4 Contact Printer Lithography

Contact printers provide basic patterning capability at the micrometer scale, with a corresponding level of overlay accuracy and reasonable throughput. While still a useful training tool, recent progress in optical direct-write technology (see below) is making these systems less relevant for lithography and will likely lead to their being less critical. They are, however, useful for aligned wafer bonding operations and obviate the need for a custom setup.

#### 10.2.5 Optical Direct Write

Optical direct-write tools enable both the fabrication of photomasks for contact printers and direct patterning of wafers. The NanoFab provides users with two tools: a dedicated mask writer and a laser writer that is suited for wafer direct write. Both tools have approximately the same resolution as a contact printer, but they obviously avoid the potential for damage and contamination associated with contact printing. The general-purpose laser writer can also perform level-to-level alignment, enabling the fabrication of more complex devices, and it operates at a throughput sufficient for small batch fabrication, obviating the need for making masks for that purpose. In addition, since there is no mask cost associated with changing designs, it enables rapid and inexpensive device design iterations.

#### 10.2.6 Nanoimprint Lithography

Given the relatively high cost of making an imprint template, and the frequent design changes that occur in a research environment, the nanoimprint system is valuable more for its ability to pattern soft materials, and thus enable studies of nanoscale material behavior, than for its potential for high-throughput pattern replication. However, the precision and accuracy of the imprint replication process is attractive for the production of standard reference materials – calibration artifacts that support the dissemination of NIST metrology.

#### 10.2.7 Soft Lithography

The ability to create patterns in soft materials, such as polydimethylsiloxane (PDMS), is important for the fabrication of micro- and nanofluidic devices, particularly for bio-related work. Molding PDMS is not compatible with other cleanroom operations, and it is performed in a separate area with controlled access to eliminate the risk of cross-contamination.

### 10.3 Etch

Once a suitable pattern has been created on the material layer of interest, it must be transferred into that layer. As noted above, our fab supports users making devices from a diverse set of materials. As is apparent from the user data shown above, we have a large community working on MEMS, and another focused on nanophotonic structures. The former requires access to deep Si etching, while the latter needs tools dedicated to the etching of III-V materials, SiN and SiO_2_ waveguides, and Si for device layers and chip separation. For both classes of users, the need for process repeatability and the requirements for control over sidewall angles and surface roughness to achieve low mechanical and optical losses mean that it is essential to enforce strict material—both mask and substrate—and process segregation. This in turn requires us to maintain both a large number of etch systems and expend considerable effort to educate users on the need for process segregation and etcher-specific material restrictions.

In a lesson learned, we realized that allowing users to create their own recipes on the etchers was contributing to the problem of maintaining process and materials segregation. In order to overcome this, minimize the burden on staff, and maximize user productivity, we deployed a set of high-quality baseline processes developed and supported by the staff. Finally, new protocols, including pre- and post-etch-chamber cleaning/conditioning steps, as well as the etch recipes, were communicated to the users by the staff, and, most importantly, enforced. In order to run a nonstandard etch recipe on a tool, a user must first demonstrate that the existing etch recipes are inadequate. If this is the case, the user and staff work together to develop a new etch process, if possible, that does not adversely affect the other processes run on that particular tool. If this is not possible, then the NanoFab manager must explain to the user that an alternative fabrication route must be pursued. This approach has eliminated the proliferation of etch recipes and has greatly improved the stability and quality of the baseline processes: Only on rare occasions are users turned away. Our etch area is now stable and producing high-quality results.

Learning the importance of process and material segregation in achieving high-quality, repeatable results, and the high number of etch systems needed to maintain this standard, was a painful lesson for the NanoFab staff and frustrating for users.

#### 10.3.1 Inductively Coupled Plasma–Reactive Ion Etch (ICP-RIE)

ICP-RIE systems provide more control over etching conditions and higher etching rates. To satisfy the needs of the MEMS community, we have two fluorocarbon-chemistry deep silicon etch tools, providing capacity and redundancy. A third ICP-RIE system is dedicated to chlorine-chemistry etching of metals, a fourth to chlorine-chemistry etching of III-Vs, and a fifth to fluorocarbon-chemistry etching of SiO_2_, SiN, and Si. As noted above, this diversity of tools is necessary to allow for sufficient process and material segregation to maintain process stability. While ICP-RIE systems are highly capable, they are complex, and the increased complexity can lead to more extended tool downtimes and challenging maintenance issues. Given that such systems cost roughly twice as much as a parallel-plate RIE system, it is important to determine the trade-offs among process capability, tool availability, and cost.

#### 10.3.2 Reactive Ion Etch (RIE)

Parallel-plate RIE tools are suitable for etching of thin dielectrics and organics. We provide two as general-purpose systems for etching tasks that do not demand tight process control. These are also used to transfer resist patterns into hard mask materials, such as SiO_2_, which enables us to eliminate the use of potentially contaminating organic masking layers in the critical ICP-RIE tools. We note here that these tools are becoming increasingly critical for our extensive nanophotonics program, which requires tight process control for SiN waveguides. This will ultimately necessitate the purchase of a dedicated tool. As a lesson learned, we have found that, even though it may appear redundant, it is important to have dedicated etch tools to support critical programs.

#### 10.3.3 Ion Milling

As noted above, our PVD operation supports the deposition of many materials, some of which, such as Fe and Ni, used for magnetic studies, or Au and Pt, used as electrode materials, have no useful volatile etch products and can therefore not be etched using reactive gas chemistries. These materials can, however, be patterned successfully using Ar ion milling. The NanoFab has an ion mill, equipped with secondary ion mass spectrometry (SIMS) end-point detection, that enables pattern transfer into a wide material set with precise control.

#### 10.3.4 HF Vapor and XeF_2_ Etching

HF vapor etching is used to selectively remove SiO_2_, while XeF_2_ removes Si, typically to release a free-standing MEMS structure. Since both of these etches take place in the gas phase, common “stiction” problems that occur when free-standing structures collapse are avoided. Again, these tools support our many MEMS fabricators.

### 10.4 Imaging and Characterization

Imaging and characterization are needed at every step of a fabrication process. Many of the more straightforward measurements are handled using the suite of small tools in the metrology and inspection bay of the NanoFab. More detailed structural and compositional characterization requires larger, more sophisticated tools. The complexity of these systems means that they require both dedicated staff support and a substantial investment in service contracts. The instruments described below are used both to support fabrication and are also used as stand-alone systems.

#### 10.4.1 Transmission Electron Microscopy (TEM)

The transmission electron microscope provides users with the ability to perform atomic-resolution imaging, and near-atomic-resolution chemical mapping *via* electron energy loss spectroscopy and energy dispersive X-ray analysis. Tomography is also available.

#### 10.4.2 Focused Ion Beam (FIB)

Focused ion beam machining is frequently used to prepare samples from specific areas for TEM imaging and analysis and can also be used for direct nanoscale patterning of almost all materials. These combined FIB/SEM tools also have the capability to provide three-dimensional reconstructions using a “slice-and-view” approach, which is used for microstructural analysis and is particularly helpful in identifying device design and process failures. The instruments are fully equipped with analytical tools such as electron backscatter diffraction (EBSD), further enhancing their utility. These tools are always in high demand, and we have two to satisfy it and provide backup.

#### 10.4.3 Scanning Electron Microscopy (SEM)

Scanning electron microscopy is indispensable for making rapid assessments of nanofabrication process performance, and it provides both qualitative and quantitative data, including compositional analysis when desired. The NanoFab has two systems capable of accommodating full-size wafers, making these instruments useful for nondestructive at-line process and device evaluation.

#### 10.4.4 X-Ray Diffractometer (XRD)

The X-ray diffractometer yields crystallographic information on thin films and can be used to determine the degree of crystallinity in thin film samples. The system has a heating stage capable of temperatures up to 1100 °C, which permits users to follow crystallographic phase changes as a function of temperature, under vacuum, air, and Ar environments. It is versatile and can be used to determine the composition of powders and polycrystalline films or the size and size distribution of nanoparticles. The layer thickness and surface and interface roughness of thin films, as well as structure, thickness, orientation, lattice mismatch, and dislocation density in epitaxial films may also be measured.

#### 10.4.5 Dynamic Light Scattering (DLS)

The dynamic light scattering instrument enables users to measure the size and size distribution of particles in suspensions, ranging from proteins a few nanometers in diameter up to inorganic particles in the micrometer range. The use of a flow-field flow-fractionation front end for the system separates particles by size before measurement, mitigating the signal deconvolution issues that can otherwise make particle size and size distribution measurements problematic.

### 10.5 Metrology

The ability to measure feature sizes, thin film thickness, stress, electrical and optical properties, surface roughness, and etch depth are all essential to enable users to characterize and control their processes, and for staff to maintain consistent tool operations and to develop processes. The NanoFab makes available scanning electron microscopes and atomic force microscopes for nanoscale dimensional metrology, interferometers and spectroscopic ellipsometers for thin-film thickness and optical properties measurements, four-point probes, a mercury probe and a parametric tester for nondestructive evaluation of material and device electronic properties, and surface and optical profilometers for topography measurement.

### 10.6 Wet Processing

Wet processing is used in all phases of device fabrication, and it frequently involves relatively hazardous chemicals. Automated systems help to ensure both process reproducibility and user safety. The NanoFab therefore maintains tools such as spin-rinse dryers and a single-wafer photoresist spray-develop tool to enhance process reproducibility, spray-acid systems for wafer cleaning, and a spray-solvent tool to minimize user contact with hazardous chemicals. Wet decks with chemistries maintained by NanoFab staff are also available for standard (*e.g.*, RCA) cleans and other processes, such as semiconductor, dielectric, and metal etching. Hoods, including dedicated solvent-only, base-only, and acid-only hoods, are available for nonstandard chemistries. Finally, the NanoFab also maintains acid neutralization and solvent collection systems for the safety of users and the environment.

### 10.7 Postprocessing

Specialty postprocessing tools are needed for further integration and include wafer scribe-and-break and dicing systems, a wire bonder, and a flip-chip bonder. Since these are not clean processes, they are housed in an area separate from the main cleanroom.

#### 10.7.1 Chemical-Mechanical Polishing (CMP)

Chemical mechanical polishing is also housed in areas separate from the main cleanroom and, while normally included with postprocessing, is better thought of as adding another way of etching materials rather than patterning them. It provides desired topographic profiles and planarization within a range of materials that cannot be processed with reactive ion etching or ion milling. As such, it enables a different set of process pathways that can resolve difficult material and process compatibility issues.

### 10.8 Ancillary Tools

Ancillary tools include items such as probe stations, optical microscopes, UV-vis spectrometers, vacuum ovens, spin-rinse dryers, hot plates, and resist spinners that support major equipment but are relatively inexpensive. For this reason, they are often overlooked, but they need to be of good quality and present in sufficient numbers so that the more expensive and critical tools that they feed can be utilized most effectively. Resist spinners can be a weak link, given their tendency to clog, especially with inexperience users. In a lesson learned, we have therefore recently purchased several spare spinners so that fresh ones can be “hot swapped” to prevent expensive tools from sitting idle when a spinner malfunctions. Similarly, we have added a small, short-cycle-time thermal evaporator to deposit Al charge dissipation layers on samples destined for electron-beam lithography. This serves to both reduce the load on the heavily subscribed PVD tools and to enable more efficient use of the electron-beam lithography systems. Modest investment in support tools can lead to significant improvements in productivity and the user experience.

## 11 Utilization and Capacity Analysis

Before discussing our capacity analysis, it is important to point out some important differences between a research fab and an industrial one. First, as noted above, the “product” mix in a research fab is extraordinarily diverse and constantly changing, by comparison with an industrial fab, making it impossible to identify a “typical” process flow. Second, in a manufacturing setting, the number and mix of tools in each area are optimized for throughput and redundancy to ensure that they are fully utilized: For example, the extremely high throughput of an optical lithography tool is matched by a suite of etchers. In a research fab, however, it is typical to have only one of each tool, resulting in an intrinsic throughput mismatch between the optical lithography and etching areas. These factors are important to consider when analyzing raw utilization data and determining what constitutes capacity.

While the high-level user data presented above help to guide the selection of tools to support the major program areas supported by the NanoFab, much more detailed information is needed to optimize facility operations. This information can help identify bottlenecks occurring because of oversubscription of tools, excessive reservation-to-tool operation ratios, or poor tool uptime. Data on cleanroom occupancy as a function of time can inform decisions on staffing levels and tool reservation policies during operating hours to reduce crowding. As noted above, estimating cleanroom capacity, and deciding what to do when nearing capacity, are difficult endeavors in the context of a research fabrication and characterization facility, such as the CNST NanoFab, that supports a diverse mix of projects, processes, and users. The constantly varying and heterogeneous nature of the devices being fabricated, along with the range of user skill levels, makes efficient utilization a constant challenge. In addition, as we note above, utilization and output are typically correlated but not identical. As an example, and lesson learned, we observed that an etcher was almost fully subscribed, clearly representing a wise investment. However, further investigation through discussions with users revealed that the reason for the heavy usage was that the etcher was highly irreproducible and that users were having to run multiple samples to obtain an acceptable result. Eventually, one user process was identified as the cause of the process instabilities, and that user was asked to find a different approach or a different facility. Once the etcher was repaired and had stabilized, the amount of utilization decreased, but overall user satisfaction and productive output increased dramatically. An important lesson learned was that it is not possible to accommodate all user requests and maintain the quality of the facilities operations.

As this example illustrates, a capacity analysis is difficult and fraught with potential pitfalls. In order to provide as complete a picture as possible, we have therefore taken two different approaches to estimate the capacity. We examine capacity as determined by cleanroom occupancy and by individual tool use. Once again, we emphasize the fact that a comprehensive flow of data is essential to making effective decisions.

### 11.1 Capacity by Cleanroom Occupancy

**Fig. 7:**
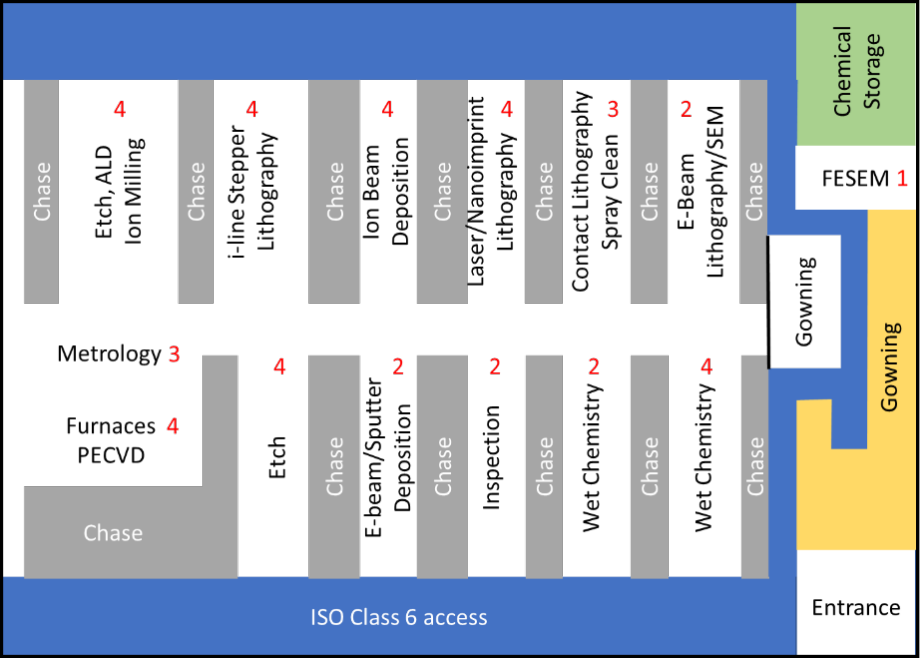
Schematic of the cleanroom with nominal maximum occupancy of each area marked in red.

The first way of determining capacity is to assess the number of people that can use the NanoFab cleanroom (as distinct from the imaging instruments or ancillary tool areas) at any given instant. Figure 7 shows the maximum occupancy possible in each area, taken to be the number of user stations, and the sum of these. The maximum total occupancy is 43, and there are typically five NanoFab staff members in the cleanroom at any one time who are occupying user stations (other staff members in the cleanroom are doing tasks that do not affect user access to the tools). The maximum possible number of users is therefore 38. For users to be able to operate efficiently and move from tool to tool to execute a process flow, we assume a maximum usable occupancy fraction of 0.75, or 29 users. We therefore define the cleanroom capacity to be 29 users.

**Fig. 8:**
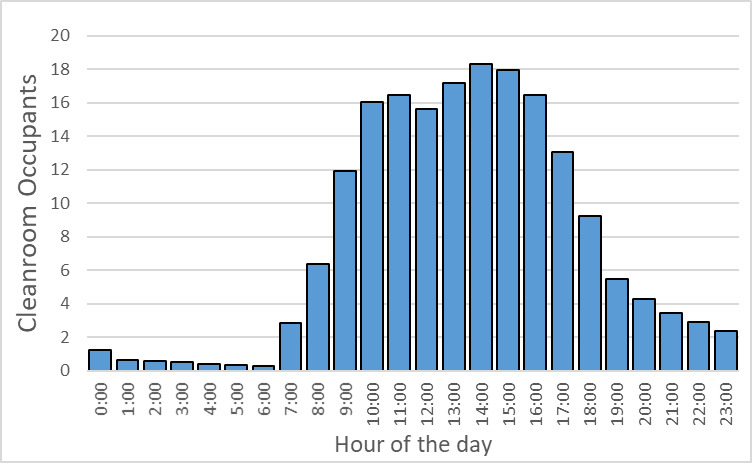
Average cleanroom occupancy by hour of the day.

**Fig. 9:**
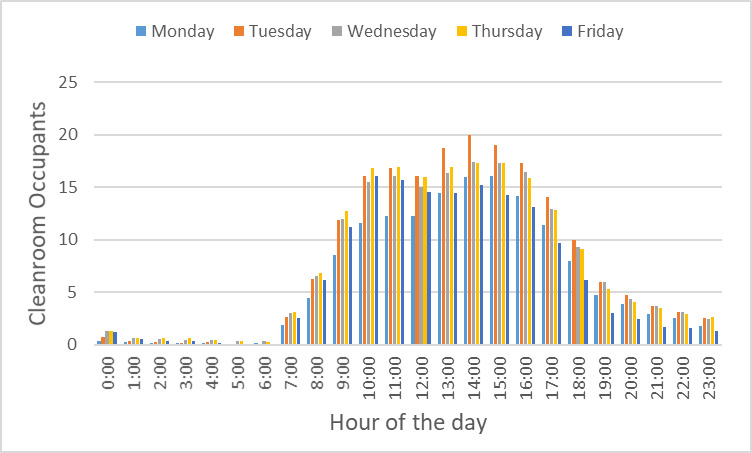
Average cleanroom occupancy by hour of the day and day of the week.

Figure 8 shows the average user (*i.e*., not including staff) cleanroom occupancy by hour, and Fig. 9 shows the average cleanroom occupancy by hour and by day of the week for the 12 months from August 28, 2017, to August 28, 2018.

**Fig. 10:**
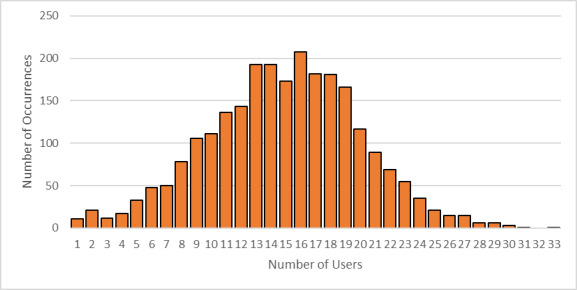
Frequency of occurrence of hourly occupancy, showing that the most common occupancy level is between roughly 13 and 18 people.

Figure 10 shows how often a given number of users is present in the cleanroom. For example, the most common number of users is 16, but the distribution has a broad peak around that number. The comparatively steep drop-off once the number of users reaches about 20 may suggest that users prefer not to come to the cleanroom when it is that busy.

**Fig. 11:**
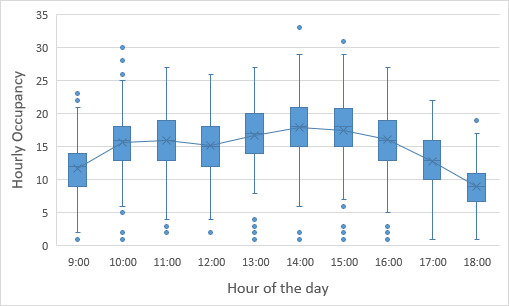
Box-and-whisker plot of hourly occupancy with first and third quartiles marked by the box, and the local minima and maxima marked by the whiskers. Outliers, marked by circles, are defined as being > 1.5× the interquartile range away from the median.

Figure 11 shows the data from Fig. 8 and Fig. 9 combined.

Although the data for peak cleanroom occupancy can be closely approximated with a normal distribution, closer inspection reveals that the distribution is slightly skewed towards lower occupancies, representing the effect of those using the weekend “buddy” system. Currently, the fraction of peak occupancies of between 15 and 22 users (50% to 75% of the maximum desirable) is ≈ 0.4, and that between 22 and 29 users (75% and 100%) is ≈ 0.06.

As utilization is increased, we can expect the distribution to become a little more skewed towards higher occupancies. However, if we continue using the normal distribution as a model, an increase in the mean peak occupancy of one standard deviation (5.1 users, or ≈ 34%) would increase the fraction of peak occupancy lying between 15 and 22 users (50% to 75% of the maximum desirable) to 0.5, but the fraction between 22 and 29 users (75% and 100%) would jump to ≈ 0.28. In such a situation, it might be desirable to implement a reservation policy that encourages users to book tools earlier or later in the day, for example, by increasing the maximum number of hours for which a reservation might be made at those times versus the middle of the day. Alternatively, the same behavior could be promoted by adopting a pricing model that charges different rates depending on time of day.

### 11.2 Capacity by Tool Availability

The second approach is to consider tool availability and utilization. The fab is only fully staffed during business hours. We determined both the total number of hours each tool is being used, and the number of hours it is used during business hours (5 days a week between 09:00 h and 18:00 h = 8 h + 1 h for lunch). As a basis for comparison, we note that there are 2250 business hours in a year (250 working days × 9 hours per day), and 8760 hours total. As discussed previously, 24/7 access is possible in principle, subject to the buddy system and site-access restrictions. Nonhazardous tools that are in general laboratory space, including the TEM, FIBs, XRD, and the external electron-beam lithography system, can be used without a buddy.

**Fig. 12:**
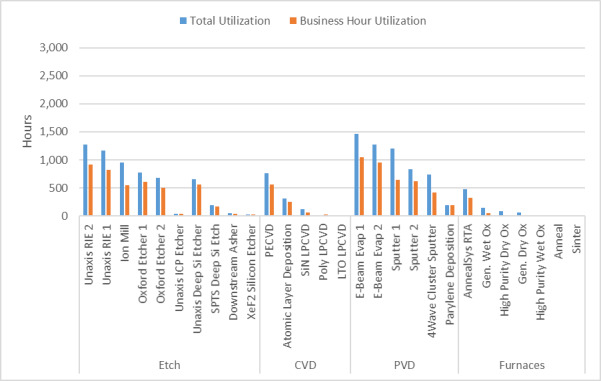
Total user hours on each tool (blue) and during business hours (orange). (Note: This data set reflects the fact that new furnaces were being installed during the time period captured here.)

**Fig. 13:**
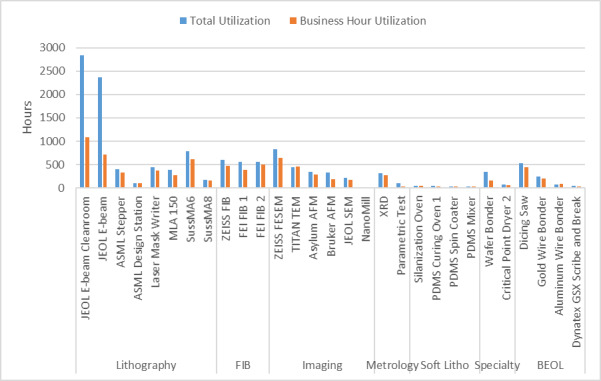
Total user hours on each tool (blue) and during business hours (orange).

The charts below (Figs. 12 and 13) show the total tool usage for all major tools. The tools most in demand reach utilization levels of approximately 50% during business hours. In most cases, there is relatively little use outside business hours, with the exception of the two electron-beam lithography systems, which have low throughput and are capable of long periods of unattended operation.

The charts (Figs. 12 and 13) illustrate one of the principal differences between a research fab and an industrial one. As noted previously, industrial fabs run a limited number of process flows, and they are optimized to maximize utilization of each tool. By contrast, a research fab must provide a collection of tools to enable as many process flows as possible (bearing in mind compatibility constraints), so no such optimization is possible, and many tools are not and cannot be fully utilized. However, it is reasonable to examine tools that are in demand and determine how to make the most efficient and effective use of them.

In addition to examining user hours on each tool, it is also important to look at all the hours logged on a tool. This data set can help to highlight tools that may be unreliable, as shown by shutdown time or excessive staff and/or vendor maintenance time. Alternatively, tools that show a large number of staff hours may be new acquisitions, and the staff may be using them to develop baseline processes, or this could indicate that a particular tool is proving problematic for users to operate, which might suggest an automation upgrade would be worthwhile.

**Fig. 14:**
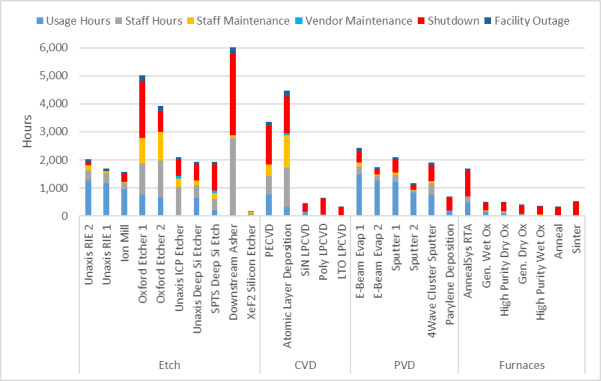
Total hours logged on each tool, broken down by category. (Note: This data set reflects the fact that new furnaces were being installed during the time period captured here.)

**Fig. 15:**
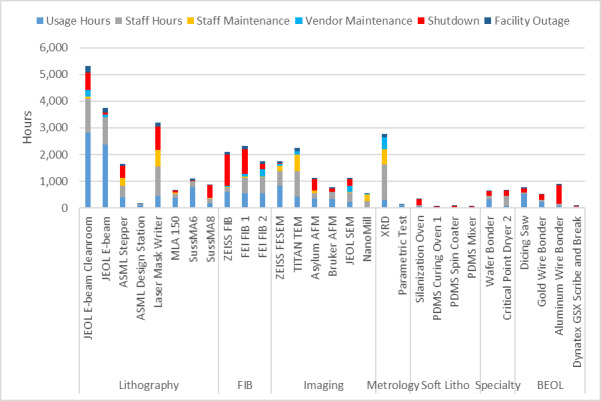
Total hours logged on each tool, broken down by category.

When looking at the mix of hours logged on each tool (Figs. 14 and 15), it is worth noting that, as illustrated in Figs. 12 and 13, most usage occurs during business hours. However, when a tool is shutdown or under staff maintenance, it is in that state around the clock, until it becomes operational again.

We illustrate the type of insight that can be gained with three examples. We first consider the downstream asher: We see that staff time and vendor maintenance time accounted for almost 3000 h apiece of activity logged on the system. This suggested that the tool was not only unreliable, requiring numerous visits by the vendor to attempt maintenance on the system, but that suitable process recipes were difficult to generate, requiring excessive staff time. Further, the function that the asher was supposed to fulfill was offloaded onto other tools, *i.e.*, RIE and ICP etchers not designed for the task, as users sought to work around the process roadblock. The end result was to derail our process segregation efforts. The lessons learned included the need to analyze staff time carefully, to understand the interconnected nature of tool usage in the fab, and the need to fix or replace underperforming tools as rapidly as possible. In addition, the specification and procurement of some tools, *e.g*., furnaces, CMP, should include a suite of processes, relevant training, and the performance metrics that must be met before tool acceptance.

A second example concerns the wafer stepper, which has a relatively small number of hours logged, which might suggest that it has minimal utility. However, this tool is designed to deliver an industrial level of throughput, so, in a research environment, a few minutes of user time can generate enough patterned wafers for many downstream process flow variations. Lastly, our newly installed and commissioned furnaces show minimal use (at the time of this writing), but the situation will change once they are released to the users.

The charts above represent a snapshot of the state of our NanoFab, but it is critical to remember that this state is continually evolving. The benefit of the constant flow of data is that positive or negative trends can be detected, and this then allows an accurate assessment of the impact of changes in everything ranging from reservation policy to maintenance schedules on the operation of each tool and on the fab as a whole.

## 12 Conclusions

In this article, we have attempted to discuss some of the more important items that should be taken into account when operating a fabrication and characterization facility, including the need for long-term vision and institutional commitment, and for the hands-on involvement of managers in facility operations. We have discussed startup, operating, and recapitalization costs, algorithms for cost recovery and tool-time allocation, and project and tool utilization data. Optimizing the operation of such a facility is a complex task and is possible only when supplied with detailed and comprehensive data. In addition, only by considering all facets of the facility’s operation can an accurate picture be developed that enables data-driven decisions that will maximize the impact of the facility. A singular focus on a metric such as user numbers or tool utilization will inevitably lead to key insights being missed. While we have developed our approach based upon the specifics of our NanoFab, we hope that the methodologies and resources presented here are useful to all those faced with this somewhat daunting mission.

## Acknowledgments

The authors would like to acknowledge Drs. David A. Czaplewski (Scientist, Center for Nanoscale Materials, Argonne National Laboratory), John Nibarger (NIST Boulder Cleanroom Manager), and Gerald Lopez (Director, Business Development Singh Center for Nanotechnology) for many helpful comments and suggestions, and Mathieu Rampant (Prometheus Computing) for his assistance with mining user data.
